# Synthesis and Biological Evaluation of 2-Phenoxyacetamide Analogues, a Novel Class of Potent and Selective Monoamine Oxidase Inhibitors

**DOI:** 10.3390/molecules191118620

**Published:** 2014-11-14

**Authors:** Wei Shen, Shian Yu, Jiaming Zhang, Weizheng Jia, Qing Zhu

**Affiliations:** 1 Department of General Surgery, Jinhua Central Hospital, Jinhua 321000, China; 2 College of Biological and Environmental Engineering, Zhejiang University of Technology, 18 Chaowang Road, Hangzhou 310014, China

**Keywords:** monoamine oxidase, inhibitors, phenoxyacetamides, inhibitory activity, cell lysates

## Abstract

Monoamine oxidases (EC 1.4.3.4; MAOs), a family of FAD-containing enzymes, is an important target for antidepressant drugs. In this paper, a series of 2-phenoxyacetamide analogues were synthesized, and their inhibitory potency towards monoamine oxidases A (MAO-A) and B (MAO-B) were evaluated using enzyme and cancer cell lysate. 2-(4-Methoxyphenoxy)acetamide (compound **12**) (SI = 245) and (2-(4-((prop-2-ynylimino)methyl)phenoxy)acetamide (compound **21**) (IC_50MAO-A_ = 0.018 μM, IC_50MAO-B_ = 0.07 μM) were successfully identified as the most specific MAO-A inhibitor, and the most potent MAO-A/-B inhibitor, respectively. The inhibitory activities of these two compounds in living cells were also further evaluated utilizing HepG2 and SHSY-5Y cell lysates.

## 1. Introduction

Monoamine oxidases (EC 1.4.3.4; MAOs) are FAD-containing enzymes that bind tightly to the outer mitochondrial membrane in the brain, liver, intestinal mucosa, and other organs and catalyze the oxidative deamination of biogenic and xenobiotic amines. There are two types of isoenzymes, MAO-A and MAO-B, which can be distinguished by their differential substrates, inhibitor selectivity, tissue distribution, and primary DNA sequences [1]. MAO-A is located predominantly in catecholaminergic neurons, while MAO-B is present in serotonergic neurons and glia. MAO-A catalyzes the oxidation of 5-hydroxytryptamine (5-HT) and norephinephrine, whereas MAO-B deaminates dopamine and 2-phenylethylamine (2-PEA). Recent studies have shown that MAOs are important target enzymes for antidepressant drugs [2]. In addition, the biotransformation of 1-methyl-4-phenyl-1,2,3,6-tetrahydropyridine (MPTP) into 1-methyl-4-phenylpyridinium (MPP+) mediated by MAO-B results in the neurodegenerative symptoms of the Parkinsonism [3]. Therefore, the study of MAO inhibitors has attracted increasing interest in recent years for their therapeutic effect on mental illness. For example, selective MAO-A inhibitors such as iproniazid, clorgyline and moclobemide exhibit antidepressant and antianxiety activity [4,5]. Selective MAO-B inhibitors such as selegiline, rasagiline and lazabemide are used as adjuncts in the treatment of Parkinson’s and Alzheimer’s diseases [6]. Unfortunately, most of the existing MAO inhibitors, such as iproniazid and tranylcypromine, have been shown to induce hepatotoxicity and another important side effect, the “cheese reaction” [7,8]. Thus, the development of new MAO inhibitors for neuro-related diseases is urgently needed.

**Figure 1:**
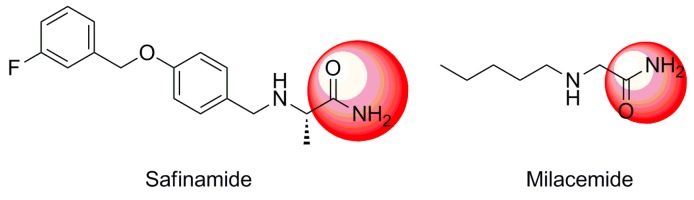
Examples of known monoamine oxidase (MAO) inhibitors bearing an acetamide group.

In order to address the need for new MAO inhibitors with fewer side effects, our group evaluated compounds previously discovered for their potential as MAOIs. Among them, we found safinamide, which was reported to be a potent anti-MAO B agent, and milacemide, which was found to be a potent MAO inhibitor and a prodrug for glycine [9,10]. Of note, both compounds possess an acetamide group (Figure 1), prompting us to hypothesize that acetamide might play an important role in inhibition of MAO activities. Therefore, we herein report the synthesis of a new series of 2-phenoxyacetamide analogues and the evaluation of their inhibition of MAO-A and MAO-B activities.

## 2. Results and Discussion

### 2.1. Chemistry

**Scheme 1:**
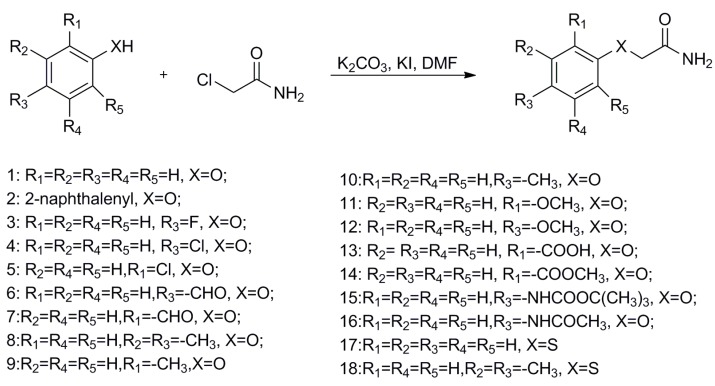
General synthetic method for 2-phenoxyacetamide analogues.

To begin, 2-phenoxyacetamide analogues were synthesized by a nucleophilic reaction, outlined in Scheme 1. Substituted phenols with electron donating (Me or OMe) or withdrawing groups (F, Cl, COOCH_3_ or CHO) reacted with 2-chloroethanamide under basic conditions, creating aryloxycarboxylic acetamides in excellent yields.

**Scheme 2:**
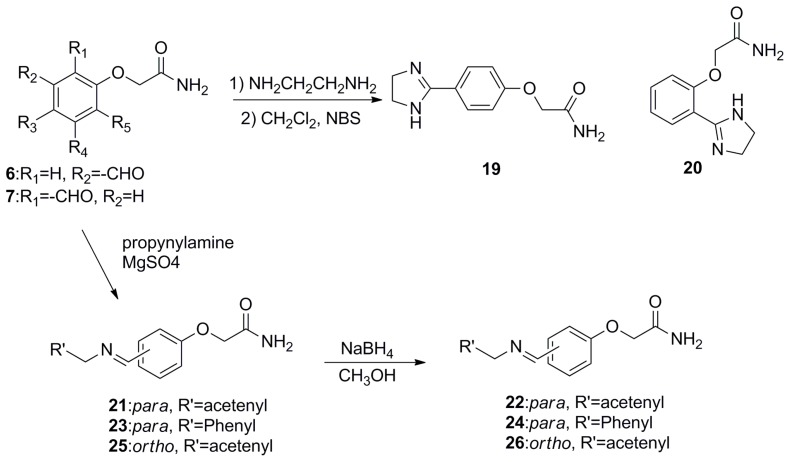
Synthetic methods for compounds **19**–**26**.

Furthermore, in order to broaden the scope of such kind of compounds, more derivatives bearing heterocycles and amines (compounds **19**–**26**) were synthesized according to the procedures outlined in Scheme 2. For example, compounds **19** and **20** were obtained in modest yield from the condensation of compound **6** or **7** with ethylenediamine. The reaction of the different aldehydes and propargylamine (or benzylamine) produced the corresponding schiff bases **21**, **23**, **25**, followed by reduction to compounds **22**, **24**, and **26** respectively, with four equivalents of NaBH_4_. Structures of target compounds **1**–**26** were confirmed by their1H and 13C NMR spectra and high-resolution electron impactmass spectra (HR-EIMS) (see SI).

### 2.2. Enzyme Inhibition Studies

All of these compounds were evaluated for their inhibitory abilities towards MAOs. The inhibition activities against MAO-A and MAO-B of the synthesized compounds were investigated by our reported method [11,12]. The IC_50_ values with MAO-A and MAO-B and the selectivity index (SI, IC_50_ of MAO-B/IC_50_ of MAO-A) of all compounds are shown in Table 1.

**Table 1:** Monamine oxidase inhibitory activity of compounds **1**–**28** ^a^.

Item	R	X	IC_50_ (μM)		SI ^b^
			MAO-A	MAO-B	
1	Phenyl	O	69	778	11.27
2	2-Naphthalenyl	O	149	542	3.64
3	4-Fluoro-phenyl	O	92	255	2.77
4	4-Chloro-phenyl	O	490	202	0.41
5	2-Chloro-phenyl	O	98	694	7.08
6	4-Formyl-phenyl	O	89	457	5.13
7	2-Formyl-phenyl	O	142	559	3.94
8	3,4-Dimethyl-phenyl	O	113	534	4.73
9	2-Methyl-phenyl	O	26	663	25.5
10	4-Methyl-phenyl	O	3	541	180
11	2-Methoxy-phenyl	O	96	775	8.07
12	4-Methoxy-phenyl	O	4	980	245
13	o-Carboxyphenyl	O	217	177	0.82
14	o-Acid ester	O	108	98	0.91
15	4-( *N*-tert-Butyl *O*-acyl)amine-phenyl	O	196	296	1.51
16	4-( *N*-acetyl)amine- phenyl	O	61	553	9.07
17	Phenyl	S	166	642	3.87
18	3,4-Dimethyl-phenyl	S	292	366	1.58
19	4-(4,5-dihydro-1H-imidazol-2-yl)-phenyl	O	61	506	8.30
20	2-(4,5-dihydro-1H-imidazol-2-yl)-phenyl	O	186	714	3.84
21	4-((Prop-2-ynylimino)methyl)-phenyl	O	0.018	0.076	4.22
22	4-((Prop-2-ynylamino)methyl)-phenyl	O	0.094	0.164	1.74
23	4-((benzylimino)methyl)-phenyl	O	96	575	5.99
24	4-((benzylamino)methyl)-phenyl	O	37	534	14.43
25	2-((Prop-2-ynylimino)methyl)-phenyl	O	0.068	0.176	2.59
26	2-((Prop-2-ynylamino)methyl)-phenyl	O	0.168	0.188	1.19
27	2-((benzylimino)methyl)-phenyl	O	147	562	3.82
28	2-((benzylamino)methyl)-phenyl	O	107	497	4.64
	Clorgyline		0.0011 (0.0014)		
	Pargyline			0.0035 (0.0038)	

^a^ Results are expressed as mean IC_50_; ^b^ SI = (IC_50_ of MAO-B)/(IC_50_ of MAO-A).

The results clearly show that almost all compounds take on higher inhibitory activity against MAO-A than MAO-B (SI > 1). Particularly, the compound **12** containing *para*-methoxyl moiety exhibits the best selectivity and the SI value is up to 245 as an MAO-A inhibitor. On the basis of the results in Table 1, the addition of methyl and methoxy groups in the benzene ring appears to increase inhibitory activity toward MAO-A rather than MAO-B. The dimethyl-substituted compound **8** significantly lost selectivity and inhibitory activity against MAO-A, compared with the single substitute compounds **9** and **10**. One can also conclude that the addition of F or Cl at the *ortho* or *para* position in the phenyl ring (compound **3**–**6**) seemed to decrease inhibitory activity against MAO-A. In order to increase inhibitory activity and selectivity, amino groups were added by modification of compounds **6** and **7** with different amines to obtain compounds **21**–**26**. Their IC_50_ values for MAO-A and B were found to be decreased by approximately 100 fold. The compound **21** bearing propynylimino moiety posses the best activity, and the IC_50_ value is up to 18 nM, which indicates that imine group may contribute to the binding affinity for MAOs. From Table 1, it can be further figured that the 2-phenoxyl derivatives **1** and **8** have better activities than the 2-thiophenyl compounds **17** and **18**, respectively, indicating that the replacement of 2-O with 2-S has very little affect on the potency of inhibition toward MAO-A and MAO-B.

Having demonstrated that 2-phenoxyacetamides displayed good inhibitory activity by enzymatic assay, we also carried out experiments to detect their inhibitory potency on the native MAOs, using lysates from human tumor cells as source of enzyme. Thus, lysates from SH-SY5Y (neuroblastoma cells) and HepG2 (human hepatocellular liver carcinoma cell) cells, where possessing only endogenous MAO-A and B activities, respectively [13,14], were utilized determine inhibitory of the newly synthesized compounds. Compounds **12**, **21**, **22**, **25** were chosen for comparison due to their excellent inhibitory activities, and the results were shown in Table 2. Substantially, all four compounds were found to be able to inhibit the native enzymes from raw samples, but display much less potent inhibitory in cell lysates than enzymes. The compound **21** was still the most potent inhibitor in SH-SY5Y and HepG2 lysates, and **21**, **22** and **25** displayed 2.3, 2.0 and 2.1 times more potent in SH-SY5Y lysate relative to HepG2 lysate, respectively, in agreement with enzyme assays. However, it is noteworthy that these three compounds significantly lost inhibitory activities in both of the lysates compared to **12**. This is most likely due to the instability of imine group in cell lysates.

**Table 2:** MAO inhibitory activity (μM) in different cell lysates ^a^.

*Enzymes/Cell Lysates*	*12*	*21*	*22*	*25*
SH-SY5Y	5.4	0.18	0.26	0.88
MAO-A	4.0	0.018	0.094	0.068
HepG2	N.D. ^b^	0.41	0.53	1.81
MAO-B	980	0.076	0.164	0.176

^a^ Results are expressed as mean IC_50_. All experiments were repeated three times; ^b^ N.D. means not determined.

### 2.3. Reversibility of Inhibition

To determine whether this new series of compounds are reversible or irreversible inhibitors toward MAOs, the reversibility of inhibition of compound **12** and Safinamide was further studied using repeated washing method. As shown in Table 3, the enzyme activities were able to be recovered by more than 80% after repeated washing, indicating that inhibition of both MAO-A and MAO-B is reversible in presence of these two compounds.

Taken together, all these findings suggest that the derivatives of acetamide may be developed as potential lead compounds for the treatment of neurodegenerative diseases (MAOI-B), and affective disorders (MAOI-A).

**Table 3:** Reversibility of MAOs inhibition with **12** and Safinamide.

Compound	MAO-A Inhibiton (%)		MAO-B Inhibiton (%)	
	Before Washing	After Washing	Before Washing	After Washing
**12** (100 nM)	88.7%	12.5%	96.9%	8.6%
Safinamide (100 nM)	36.1%	9.8%	91.3%	7.1%

## 3. Experimental Section

### 3.1. Materials and Methods

Silica gel (100–200 mesh, Qingdao Haiyang Chemical Co., Qingdao, China) was used for flash column chromatography. Analytical thin-layer chromatography was performed with GF254 silica gel. ^1^H-NMR spectra were recorded on a Bruker Avance 300 MHz apparatus. HR-ESI-MS and HR-EI analysis were measured on a Bruker micrOTOF-QII and Waters GCT Premier GC-TOF-MS spectrometer, respectively. All chemicals were purchased from the Eastern China Chemical Co (Hangzhou, China). All fluorescent data was recorded on Spectrum M2 (Molecular Device Company). Human recombinant Monoamine Oxidase A (M7316) and B (M7441) (5 mg/mL) were purchased from Sigma Aldrich. Pierce BCA protein assay kit (23227) was purchased from Thermo Scientific.

### 3.2. General Procedure for the Synthesis of Derivatives of Acetamides [15]

#### 3.2.1. General Procedure for the Preparation of Compounds **1–18**

2-Chloroethanamide (1 mmol), K_2_CO_3_ (1.5 mmol) and KI (0.1 mmol) was added to a solution of phenol (1 mmol) in dry DMF. The reaction mixture was stirred at room temperature for 5 h, and was extracted with EtOAc twice. The organic layer was dried over anhydrous MgSO_4_, and the solvent stripped out in a rotary evaporator to obtain the crude product, which was further purified using silica gel chromatography to obtain a white solid.

Compound **1**: ^1^H-NMR (CDCl_3_-*d*_3_): δ 4.57 (s, 2H), 5.95 (brs, 1H), 6.71 (brs, 1H), 6.96 (d, *J =* 7.8 Hz, 2H), 7.06 (t, *J =* 7.3 Hz, 1H), 7.35 (m, 2H). ^13^C-NMR (CDCl_3_-*d*_3_): δ 67.9, 113.7, 123.5, 129.4, 158.1, 171.6. HR-MS (EI) calculated for C_8_H_9_NO_2_ [M^+^] 151.0633 found 151.0628.

Compound **2**: ^1^H-NMR (DMSO-*d*_6_): δ 4.71 (s, 2H), 5.97 (brs, 1H), 6.61 (brs, 1H), 6.88 (d, *J =* 8.0 Hz, 1H), 7.45 (m, 4H), 7.88 (m, 1H), 8.39 (m, 1H). ^13^C-NMR (DMSO-*d*_6_): δ 67.7, 106.2, 110.7, 122.8, 124.5, 125.7, 126.0, 126.5, 127.3, 134.4, 153.1, 169.5. HR-MS (EI) calculated for C_12_H_11_NO_2_ [M^+^] 201.0790 found 202.0775.

Compound **3**: ^1^H-NMR (DMSO-*d*_6_): δ 4.82 (s, 2H), 5.99 (s, 2H), 6.92 (s, 2H), 7.78 (m, 2H) 8.11 (m, 2H). ^13^C-NMR (DMSO-*d*_6_): δ 68.1, 115.2 (*J* = 6.9 Hz), 116.5 (*J* = 23.2 Hz), 152.4, 155.1, 171.6 (*J* = 238.1 Hz). HR-MS (EI) calculated for C_8_H_9_FNO_2_ [M^+^] 170.0617 found 170.0602.

Compound **4**: ^1^H-NMR (DMSO-*d*_6_): δ 4.75 (s, 2H), 6.18 (s, 2H), 6.88 (m, 4H) 7.24 (m, 2H). ^13^C-NMR (DMSO-*d*_6_): δ 68.1, 115.7, 117.1, 132.4, 154.7, 171.4. HR-MS (EI) calculated for C_8_H_9_ClNO_2_ [M^+^] 185.0244, 187.0214, found 185.0236, 187.0221.

Compound **5**: ^1^H-NMR (DMSO-*d*_6_): 4.75 (s, 2 H), 6.12, 6.35 (s, 2H), 6.99 (m, 2 H), 7.32 (m, 3H). ^13^C-NMR (DMSO-*d*_6_): δ 68.1, 122.1, 122.6, 129.3, 132.4, 170.4. HR-MS (EI) calculated for C_8_H_9_ClNO_2_ [M^+^] 185.0244, 187.0214, found 185.0250, 187.0238.

Compound **6**: ^1^H-NMR (DMSO-*d*_6_): δ 4.74 (s, 2 H), 6.00 (brs, 1H), 6.52 (brs, 1H), 7.00 (d, *J =* 7.8 Hz, 2H), 7.89 (d, *J =* 7.8 Hz, 2H), 9.93 (s, 1H). ^13^C-NMR (DMSO-*d*_6_): δ 68.5, 115.6, 131.0, 133.5, 164.2, 191.3. HR-MS (EI) calculated for C_9_H_9_NO_3_ [M^+^] 179.0582, found 179.0584.

Compound **7**: ^1^H-NMR (DMSO-*d*_6_): δ 4.75 (s, 2 H), 6.00 (brs, 1H), 6.52 (brs, 1H), 7.54 (m, 4H), 9.88 (s, 1H). ^13^C-NMR (DMSO-*d*_6_): δ 69.7, 115.2, 122.9, 126.1, 133.5, 136.6, 163.2, 170.7. HR-MS (EI) calculated for C_9_H_9_NO_3_ [M^+^] 179.0582, found 179.0579.

Compound **8**: ^1^H-NMR (CDCl_3_-*d*_3_): δ 2.31 (s, 3H), 2.37 (s, 3H), 7.58 (s, 2H), 5.67 (brs, 1H), 6.62 (brs, 1H), 7.07 (m, 3H). ^13^C-NMR (CDCl_3_-*d*_3_): δ 18.1, 19.6, 69.2, 1112.3, 115.9, 128.6, 129.9, 140.3, 157.6, 170.3. HR-MS (EI) calculated for C_10_H_13_NO_2_ [M^+^] 179.0946, found 179.0934.

Compound **9**: ^1^H-NMR (CDCl_3_-*d*_3_): δ 2.18 (s, 3H), 4.58 (s, 2H), 5.67 (brs, 1H), 6.62 (brs, 1H), 7.12 (m, 4H). ^13^C-NMR (CDCl_3_-*d*_3_): δ 15.6, 69.6, 113.5, 123.1, 126.7, 1267.2, 132.9, 157.3, 169.8. HR-MS (EI) calculated for C_9_H_11_NO_2_ [M^+^] 115.0790, found 115.0779.

Compound **10**: ^1^H-NMR (CDCl_3_): δ 2.28 (s, 3H), 4.58 (s, 2H), 5.61 (brs, 1H), 6.60 (brs, 1H), 6.81 (d, *J =* 8.5 Hz, 2H), 7.08 (d, *J = * 8.5 Hz, 2H). ^13^C-NMR (CDCl_3_-*d*_3_): δ 55.7, 68.0, 114.7, 115.8, 151.4, 154.1, 171.6. HR-MS (EI) calculated for C_9_H_11_NO_2_ [M^+^] 165.0790, found 165.0802.

Compound **11**: ^1^H-NMR (DMSO-*d*_6_): δ 3.92 (s, 3H), 4.57 (s, 2H), 5.95 (brs, 2H), 7.00 (m, 4H). ^13^C-NMR (DMSO-*d*_6_): δ 55.3, 109.2, 116.7, 120.6, 122.3, 146.9, 150.5, 170.1. HR-MS (EI) calculated for C_9_H_11_NO_3_ [M^+^] 181.0739, found 181.0742.

Compound **12**: ^1^H-NMR (DMSO-*d*_6_): δ 3.97 (s, 3H), 4.57 (s, 2H), 5.81 (brs, 1H), 6.70 (brs, 1H), 6.93 (m, 4H). ^13^C-NMR (DMSO-*d*_6_): δ 20.4, 67.4, 69.2, 114.5, 130.2, 131.5, 155.2, 171.5. HR-MS (EI) calculated for C_9_H_11_NO_3_ [M^+^] 181.0739, found 181.0721.

Compound **13**: ^1^H-NMR (DMSO-*d*_6_): δ 4.74 (s, 2H), 5.67 (brs, 1H), 6.62 (brs, 1H), 7.50 (m, 4H), 12.09 (brs, 1H). ^13^C-NMR (DMSO-*d*_6_): δ 69.6, 115.2, 117.2, 122.1, 132.6, 136.5, 158.4, 166.2, 170.3. HR-MS (EI) calculated for C_9_H_9_NO_4_ [M^+^] 195.0532, found 195.0526.

Compound **14**: ^1^H-NMR (DMSO-*d*_6_): δ 3.38 (s, 3H), 4.78 (s, 2H), 5.67 (brs, 1H), 6.62 (brs, 1H), 7.49 (m, 4H), 10.09 (brs, 1H). ^13^C-NMR (DMSO-*d*_6_): δ 51.2, 69.6, 114.9, 117.1, 122.5, 132.6, 136.5, 158.8, 166.0, 170.3. HR-MS (EI) calculated for C_10_H_11_NO_4_ [M^+^] 209.0688, found 209.0697.

Compound **15**: ^1^H-NMR (DMSO-*d*_6_): δ 1.40 (s, 9H), 4.75 (s, 2H), 7.38 (m, 4H). ^13^C-NMR (DMSO-*d*_6_): δ 28.9, 69.6, 79.2, 112.5, 120.3, 122.6, 127.7, 148.2, 148.9, 153.0, 170.1. HR-MS (EI) calculated for C_13_H_18_N_2_O_4_ [M^+^] 266.1267, found 266.1258.

Compound **16**: ^1^H-NMR (DMSO-*d*_6_): δ 2.11 (s, 3H), 4.76 (s, 2H), 7.03 (m, 4H). ^13^C-NMR (DMSO-*d*_6_): δ 25.1, 70.2, 113.1, 120.9, 121.6, 126.8, 129.5, 169.6, 170.1. HR-MS (EI) calculated for C_10_H_12_N_2_O_3_ [M^+^] 208.0848, found 208.0857.

Compound **17**: ^1^H-NMR (CDCl_3_-*d*_3_): δ 3.63 (s, 2H), 6.10 (brs, 1H), 6.70 (brs, 1H), 7.27 (m, 4H). ^13^C-NMR (CDCl_3_-*d*_3_): δ 38.2, 127.3, 130.1, 130.3, 136.8, 174.3. HR-MS (EI) calculated for C_8_H_9_NOS [M^+^] 167.0405, found 167.0412.

Compound **18**: ^1^H-NMR (CDCl_3_-*d*_3_): δ 2.30 (s, 3H), 2.38 (s, 3H), 4.58 (s, 2H), 5.67 (brs, 1H), 6.62 (brs, 1H), 7.03 (m, 3H), ^13^C-NMR (CDCl_3_-*d*_3_): δ 21.9, 38.2, 127.6, 131.9, 135.2, 174.9. HR-MS (EI) calculated for C_9_H_11_NOS [M^+^] 181.0561, found 181.0554.

#### 3.2.2. Preparation of Compounds **19** and **20**

Ethane-1,2-diamine (1.10 mmol) was added to a solution of compounds **6** or **7** (1 mmol) in dry CH_2_Cl_2_. The reaction mixture was stirred at room temperature for 40 min, then NBS (1 mmol) was added and the reaction was stirred overnight. The resultant precipitate was extracted with chloroform and washed successively with water. The organic layer was dried over anhydrous sodium sulfate, and the solvent stripped out in a rotary evaporator to yield the crude product. The residue was further purified using silica gel chromatography to obtain compound **19** or **20**.

Compound **19**: ^1^H-NMR (DMSO-*d*_6_): δ 3.80 (s, 4H), 3.96 (brs, 1H), 4.82 (s, 2H), 5.97 (brs, 1H), 6.82 (brs, 1H), 6.91 (d, *J =* 8.8 Hz, 2H), 7.77 (d, *J =* 8.8 Hz, 2H). ^13^C-NMR (DMSO-*d*_6_): δ 46.9, 71.5, 112.5, 130.2, 157.2, 164.8, 167.5. HR-MS (EI) calculated for C_11_H_13_N_3_O_2_ [M^+^] 219.1008, found 219.1023.

Compound **20**: ^1^H-NMR (DMSO-*d*_6_): δ 3.80 (m, 4H), 4.83 (s, 2H), 5.67 (brs, 1H), 6.62 (brs, 1H), 7.04 (m, 2H), 7.43 (m, 1H), 8.00 (m, 1H). ^13^C-NMR (DMSO-*d*_6_): δ 46.9, 71.5, 110.1, 112.9, 120.6, 128.4, 132.0, 157.2, 164.8, 167.5. HR-MS (EI) calculated for C_11_H_13_N_3_O_2_ [M^+^] 219.1008, found 219.1018.

#### 3.2.3. Preparation of Compounds **21**, **23** or **25**

Propargylamine or benzylamine (1.1 mmol) and MgSO_4_ (0.2 g) were added to a solution of compound **6** or **7** (1 mmol) in dry THF (10 mL). The reaction mixture was stirred at room temperature for 4 h. After completion of the reaction, the solid was removed by filtration and the solvent was distilled, followed by the addition of water and EtOAc. The organic layer was dried over MgSO_4_, filtered, and the solvent removed under vacuum. The residue was chromatographed to obtain the products **21**, **23** or **25**.

Compound **21**: ^1^H-NMR (DMSO-*d*_6_): δ 2.83 (s, 1H), 4.22 (m, 2H), 4.59 (s, 2H), 5.18 (m, 2H), 5.82 (brs, 1H), 6.50 (brs, 1H), 6.89 (d, *J = * 8.9 Hz, 2H), 7.08 (d, *J =* 8.9 Hz, 2H), 8.61 (s, 1H). ^13^C-NMR (DMSO-*d*_6_): δ 48.2, 67.7, 75.2, 79.1, 113.0, 134.0, 132.5, 171.2, 160.6, 190.2. HR-MS (EI) calculated for C_12_H_12_N_2_O_2_ [M^+^] 216.0899, found 216.0873.

Compound **23**: ^1^H-NMR (DMSO-*d*_6_): δ 4.50 (s, 2H), 4.77 (s, 2H), 5.85 (brs, 1H), 6.56 (brs, 1H), 6.92 (d, *J =* 8.5 Hz, 2H), 7.28 (m, 5H), 7.72 (d, *J =* 8.5 Hz, 2H), 8.31 (s, 1H). ^13^C-NMR (DMSO-*d*_6_): δ 65.1, 69.9, 115.1, 125.2, 125.8, 126.7, 127.2, 128.4, 129.0, 131.3, 138.9, 161.0, 162.8, 170.3. HR-MS (EI) calculated for C_16_H_16_N_2_O_2_ [M^+^] 268.1212, found 268.1225.

Compound **25**: ^1^H-NMR (DMSO-*d*_6_): δ 2.80 (s, 1H), 4.36 (s, 2H), 4.65(s, 2H), 6.92 (m, 2H), 7.35 (m, 1H), 7.89 (m, 1H), 8.65 (s, 1H). ^13^C-NMR (DMSO-*d*_6_): δ 44.8, 70.3, 71.1, 81.2, 114.4, 121.2, 130.6, 132.3, 132.3, 142.5, 165.5, 190.6. HR-MS (EI) calculated for C_12_H_12_N_2_O_2_ [M^+^] 216.0899, found 216.0908.

#### 3.2.4. Preparation of Compound **22**, **24** or **26**

Compound **21**, **21** or **25** (1 mmol) and NaBH_4_ (3 mmol) were dissolved in absolute EtOH under mechanical stirring for 15 min. The ethanolic filtrate was evaporated to dryness, dissolved in DCM, and washed with water. The organic layer was dried over MgSO_4_, filtered, and evaporated to dryness to obtain compounds **22**, **24** or **26**.

Compound **22**: ^1^H-NMR (DMSO-*d*_6_): δ 2.65 (m, 1H), 3.72 (s, 2H), 4.65(s, 2H), 6.86 (d, *J =* 8.6 Hz, 2H), 7.24 (d, *J = * 8.6 Hz, 2H). ^13^C-NMR (CDCl_3_-*d*_3_): δ 38.8, 52.6, 68.1, 72.8, 83.4, 113.6, 132.1, 131.0, 156.9, 169.2. HR-MS (EI) calculated for C_12_H_14_N_2_O_2_ [M^+^] 218.1055, found 218.1070.

Compound **25**: ^1^H-NMR (DMSO-*d*_6_): δ 2.80 (s, 1H), 4.36 (s, 2H), 4.65(s, 2H), 6.92 (m, 2H), 7.35 (m, 1H), 7.89 (m, 1H), 8.65 (s, 1H). ^13^C-NMR (DMSO-*d*_6_): δ 44.8, 70.3, 71.1, 81.2, 114.4, 121.2, 130.6, 132.3, 132.3, 142.5, 165.5, 190.6. HR-MS (EI) calculated for C_12_H_12_N_2_O_2_ [M^+^] 216.0899, found 216.0908.

Compound **26**: ^1^H-NMR (DMSO-*d*_6_): δ 2.65 (s, 1H), 3.47 (m, 2H), 3.78 (s, 2H), 4.85 (s, 2H), 5.79 (brs, 1H), 6.64 (brs, 1H), 6.90 (m, 1H), 7.23 (m, 3H). ^13^C-NMR (CDCl_3_-*d*_3_): δ 39.7, 46.9, 69.3, 72.1, 83.4, 112.4, 119.8, 123.1, 127.6, 128.2, 157.6, 169.5. HR-MS (EI) calculated for C_12_H_14_N_2_O_2_ [M^+^] 218.1055, found 218.1050.

### 3.3. Biochemistry

#### 3.3.1. MAOs Inhibitory Assay

MAO IC_50_ values were determined using our reported fluorescence-based microplate assay [11]. Briefly, MAO-A (or -B) and inhibitors (10 nM–1000 μM) were incubated in borate buffer (50 mM, pH 8.4) at 37 °C for 3 h, followed by the addition of 50 μM 4-methyl-7-(3-aminopropoxy) coumarin (Km 62 μM or 82 μM for MAO-A and MAO-B, respectively). The fluorescent signals were measured at λ_ex_ 360 nm and λ_em_ 460 nm on a Spectrum-M2 spectrofluorometer.

IC_50_ values were calculated by nonlinear regression analysis using the four-parameter equation [Y = max − (max − min)/(1 + (X/IC_50_)^N^] where Y is the observed fluorescence, X is the concentration of inhibitor, max is the fluorescence in the absence of inhibitor (0% control), min is the fluorescence in the presence of inhibitor (MAO activity was completed inhibited, 100% control), IC_50_ is the concentration of inhibitor that gave 50% increase in fluorescence, and N is the empirical Hill slope. Curve fitting was performed using commercial software (IDBS ActivityBase XE). Each IC_50_ was calculated based on 5 points ranged from 10 nM to 1 mM (10, 20, 40, 80 nM, 0.1, 0.2, 0.4, 0.8, 1, 2, 4, 8, 20, 40, 80, 200, 400, 800, 1000 μM) based on its value of IC_50_.

#### 3.3.2. Cell Lysates Preparation

Cells were cultured at 37 °C and 5% CO_2_ in RPMI-1640 media containing 100 units/mL penicillin, 100 μg/mL streptomycin, and 10% FBS. For lysis, cells were resuspended at 5 × 10^7^ cells/mL in lysis buffer (42.3 mM PBS, 126 mM NaCl, 1.27 mM MgCl_2_, 0.85 mM EDTA, 1 mM PMSF, 0.95% Triton X-100, 9.5% glycerol, 4% 25× complete protease inhibitor, pH 7.4). The cells were vortexed to mix and keep on ice for 30 min, and then sonicated to break the cells (180 watts). The cell lysate was then clarified by ultracentrifugation at 4 °C for 30 min at 45,000. Total protein concentration was determined via a Bradford Protein Assay Kit (Bio-Rad) and cell lysates were stored at −80 °C until further use.

#### 3.3.3. Reversibility Experiments

Reversibility of the MAO inhibition with derivatives of acetamides was evaluated by a reported method [16]. MAO-A or B were incubated with 21 (50 nM) or safinamide (100 nm) as the reference in a sodium phosphate buffer (PBS, 50 mM, pH 7.4) at 37 °C. After incubating for 2 h, an aliquot was stored at 4 °C for the measurement of MAO-A and -B activity. The remaining reaction solution was placed in an Ultrafree-0.5 centrifugal tube with a 30 kDa Biomax membrane (Millipore) and centrifuged at 9000 *g* for 20 min at 4 °C. The enzyme retained in the membrane was resuspended in a sodium phosphate buffer and centrifuged two successive times. Then, the enzyme retained in the membrane was resuspended in PBS (100 mL) and an aliquot of this suspension was used for MAO-A and -B activity measurement. In order to define 100% MAO activity, control experiments were performed simultaneously by replacing the inhibitors with appropriate dilutions of the vehicles. The corresponding values of percent MAO isoforms inhibition (with and without repeated washing) were calculated based on the control experiments.

## 4. Conclusions

In conclusion, we have synthesized a series of 2-phenoxyacetamide analogues and evaluated their monoamine oxidase (MAO) A/B inhibitory activity and selectivity *in vitro*. The results show that most of the synthesized compounds are potent and selective inhibitors of MAO-A rather than of MAO-B. Of particular importance is compound **12**, which has exhibited excellent inhibitory activity selectivity toward MAO-A, and compound **21**, which displayed the highest MAO-A inhibitory potency. Future studies aimed at designing new MAO inhibitors should focus on addition of acetamide, which we have established as a unique functional group capable of potently inhibition MAOs.
